# 

**Published:** 1890-01

**Authors:** 


CONTENTS.

Vol. 37 No. 1.

| | PAGE. |
|---|---|
| The New Year | 1 |
| Looking Forward | 2 |
| Exercise and Cleanliness | 6 |
| Trichinosis | 9 |
| Home Training of Girls | 11 |
| A Queen's Prescription | 12 |
| The Feet | 15 |
| Ventilation | 16 |
| The Christian Buddha | 17 |
| A Hygienic Necessity | 17 |
| The Effects of Tight Clothing | 18 |
| The Care of Infants | 19 |
| Letting Babies walk Too Early | 20 |
| The Laws of Health | 20 |
| The Walled Lake | 21 |
| Household | 21 |
| Miscellaneous | 22 |
| Literary | 23 |

INDEX TO ADVERTISEMENTS OF HALF A PAGE AND OVER.

| | PAGE. |
|---|---|
| An Unprecidented Offer | I |
| Celluloid | II |
| Hollow Suppositories | III |
| Christ Before Pilate | IV |
| Lactopeptine | V |
| E. C. Morris & Co | VI |
| Dennin's Certain Cure | VII |
| Everybody's Typewriter | VIII |
| The Herb Cure Co | X |
| Revised Premium List | XI |

